# DNA methylome profiling of granulosa cells reveals altered methylation in genes regulating vital ovarian functions in polycystic ovary syndrome

**DOI:** 10.1186/s13148-019-0657-6

**Published:** 2019-04-11

**Authors:** Pooja Sagvekar, Pankaj Kumar, Vijay Mangoli, Sadhana Desai, Srabani Mukherjee

**Affiliations:** 1 Department of Molecular Endocrinology, ICMR-National Institute for Research in Reproductive Health, J.M. Street, Parel, Mumbai, Maharashtra 400012 India; 2 Colin Jamura Lab, Institute for Stem Cell Biology and Regenerative Medicine (inStem), National Centre for Biological Sciences (NCBS), GKVK, Bellary Road, Bangalore, 560065 India; 3 Fertility Clinic and IVF Center, 12-Springfield, 19-Vachha Gandhi Road, Gamdevi, Mumbai, Maharashtra 400007 India

**Keywords:** PCOS, Methylome profiling, DNA methylation, IVF, Granulosa cells, NGS

## Abstract

**Background:**

Women with polycystic ovary syndrome (PCOS) manifest a host of ovarian defects like impaired folliculogenesis, anovulation, and poor oocyte quality, which grossly affect their reproductive health. Addressing the putative epigenetic anomalies that tightly regulate these events is of foremost importance in this disorder. We therefore aimed to carry out DNA methylome profiling of cumulus granulosa cells and assess the methylation and transcript expression profiles of a few differentially methylated genes contributing to ovarian defects in PCOS. A total of 20 controls and 20 women with PCOS were selected from a larger cohort of women undergoing IVF, after carefully screening their sera and follicular fluids for hormonal and biochemical parameters. DNA extracted from cumulus granulosa cells of three women each, from control and PCOS groups was subjected to high-throughput, next generation bisulfite sequencing, using the Illumina HiSeq 2500® platform. Remaining samples were used for the validation of methylation status of some identified genes by pyrosequencing, and the transcript expression profiles of these genes were assessed by quantitative real-time PCR.

**Results:**

In all, 6486 CpG sites representing 3840 genes associated with Wnt signaling, G protein receptor, endothelin/integrin signaling, angiogenesis, chemokine/cytokine-mediated inflammation, etc., showed differential methylation in PCOS. Hypomethylation was noted in 2977 CpGs representing 2063 genes while 2509 CpGs within 1777 genes showed hypermethylation. Methylation differences were also noted in noncoding RNAs regulating several ovarian functions that are dysregulated in PCOS. Few differentially methylated genes such as aldo-keto reductase family 1 member C3, calcium-sensing receptor, resistin, mastermind-like domain 1, growth hormone-releasing hormone receptor and tumor necrosis factor, which predominantly contribute to hyperandrogenism, premature luteolysis, and oocyte development defects, were explored as novel epigenetic candidates in mediating ovarian dysfunction. Methylation profiles of these genes matched with our NGS findings, and their transcript expression patterns correlated with the gene hypo- or hypermethylation status.

**Conclusion:**

Our findings suggest that the epigenetic dysregulation of genes involved in important processes associated with follicular development may contribute to ovarian defects observed in women with PCOS.

**Electronic supplementary material:**

The online version of this article (10.1186/s13148-019-0657-6) contains supplementary material, which is available to authorized users.

## Background

Polycystic ovary syndrome (PCOS), one of the leading causes of anovulatory infertility, is characterized by ovarian, neuroendocrine, and metabolic perturbations in women of reproductive age group. With a global prevalence of 6–15% [1], it generally features irregularity or absence of menses and presence of multiple ovarian cysts on ultrasonography, in addition to hyperandrogenemia and systemic insulin excess. Increased pulsatility of GnRH neurons at the hypothalamo-hypophyseal interface elevates gonadotropic secretion of LH over FSH, which alongside co-gonadotropic actions of insulin, promotes increased production of androgens from ovarian folliculo-thecal cells [2, 3]. Adding to the pool of free androgens in circulation is the insulin-mediated suppression of hepatic sex hormone binding globulin (SHBG), which increases the bioavailability of testosterone, thereby affecting its clearance [4]. The cumulative actions of all these events trigger a series of physiological defects including ovarian cyst formation, amenorrhoea, anovulation, infertility, hyperandrogenism, insulin resistance, hyperinsulinemia, obesity, glucose intolerance, lipid abnormalities, type 2 diabetes mellitus (T2DM), hypertension, and cardiovascular disease. In parallel, there is an intrinsic elevation of anti-Mullerian hormone (AMH) due to the presence of cystic follicles arrested in preantral to antral stages [5]. These key events dictate the principal dogma behind the pathophysiology of PCOS understood so far.

Although genetic factors impacting the development and progression of PCOS have been amply investigated, identification and experimental corroboration of cognate epigenetic factors that may contribute to the pathophysiology of this multifaceted disorder remain enigmatic. Both environmental and physiological factors serve as strong determinants for epigenetic alterations. Factors such as intrinsic hormonal aberrations, dysregulation of intrauterine milieu by endocrine disruptors (EDCs) during gestational periods, and lifestyle modifications in subsequent phases of growth and development have been recently implicated in epigenetic predisposition to this disease. Pioneering investigations on epigenetic alterations in PCOS began with studies on peripheral blood leukocytes (PBLs) [6]. However, tissue specificity of epigenetic modifications renders it difficult to extrapolate epigenetic data derived from circulating cells like PBLs, to organs such as ovaries or adipose tissues that are highly affected in PCOS [7]. This necessitates the undertaking of clinical epigenetic studies at a tissue-specific level. Ovary withstands most of the hormonal assaults triggered by systemic aberrations in the neuroendocrine-ovarian axis. It is therefore a primary hotspot for epigenetic perturbations, which may contribute to the multiple follicular and oocyte defects observed in women with PCOS. Defects related to steroidogenesis, follicular growth and dominance, ovulation, oocyte developmental competence, cumulus-oophorus complex (COC) expansion, luteal maintenance, etc., which are under stringent control of gonadotropins and other hormones, have been well documented so far. Therefore, identification of locus/gene-specific epigenetic alterations in the ovaries of these women is of prime significance to understand the pathophysiology of this disorder.

So far, few studies conducted to identify global DNA methylation differences in PBLs and mural as well as cumulus granulosa cells (CGCs) of women with PCOS have yielded ambiguous findings [6, 8, 9]. Also, promoter methylation profiles of a few established candidate genes of PCOS including yes-associated protein (*YAP1*), follistatin (*FST*), aromatase (*CYP19A1*) and  luteinizing hormone chorionic gonadotropin receptor (LHCGR) have been investigated in these cells, and ovarian tissues by some groups till date [10–14]. Among these genes, *LHCGR* has been consistently reported to be hypomethylated in women with PCOS and in animal models of PCO [14, 15]. Subsequently, a few high-throughput attempts were made to identify some differentially methylated genes (DMGs) in women with PCOS. These included the use of diverse approaches such as methylated DNA immunoprecipitation (MeDIP) [16] and Illumina platform-based methylation microarray [17] in peripheral blood leukocytes; MeDIP coupled with methyl promoter enrichment microarray [18], as well as methylation microarray combined with microarray-based transcriptome analysis [19] in ovarian tissue biopsies and adipose tissue samples [20]; total RNA sequencing (RNA-seq) coupled with methylation measured by base cleavage and mass spectrometry (EpiTYPER) [21]; and lastly, methylation microarray in mural granulosa cells (MGCs) collected from women undergoing controlled ovarian hyperstimulation (COH) [22]. However, next generation sequencing (NGS)-based methylome studies spanning individual CpG sites, specifically in CGCs which participate in extensive cross talk between the developing oocyte and surrounding follicular milieu while facilitating meiotic maturation and ovulation of competent oocytes, are yet to be reported in women with PCOS. In this study, we have carried out comparative genome-wide bisulfite sequencing of CGCs obtained from women with PCOS and normovulatory healthy controls, using a NGS-based, multiplexed Methyl-Capture Sequencing (MC-Seq) approach. MC-Seq is advantageous over reduced representative bisulfite sequencing (RRBS) and MeDIP-Seq, in that it avoids the over-representation of recurring reads and also over Infinium 450 K methyl-microarray wherein it enables the user to opt for much greater coverage of the epigenome (3.4 million CpG sites and 20.8 million non-CpG sites, as opposed to 4,50,000 CpG sites and 3091 non-CpG loci, respectively) [23]. Also, Infinium microarrays target only those genes that are known to be differentially methylated in some cancers while MC-Seq differentiates between any genomic region bearing altered methylation marks. Additionally, compared to whole-genome bisulfite sequencing (WGBS), MC-Seq is cost effective and can identify novel genomic loci while reducing the processing time associated with WGBS. This study provides insights on genome-wide data on CpG sites that show altered methylation at a single base resolution in CGCs of women with PCOS.

## Results

### Hormonal characterization and oocyte quality parameters of the study participants

All study participants were subjected to stringent anthropometric, hormonal, and biochemical characterization prior to further investigation (Table 1). Baseline hormonal and biochemical profiling of 20 controls and 20 women with PCOS (days 3–5 of the follicular phase of menstrual cycle) showed that the baseline levels (day 3 follicular phase estimates in serum) of luteinizing hormone (LH), LH/follicle-stimulating hormone (FSH) ratio, and of AMH were high, while the levels of FSH were low in women with PCOS, compared to controls. Prolactin and thyroid-stimulating hormone (TSH) levels were similar in both groups. Serum levels of estradiol (E_2_) and progesterone (P_4_) measured before the administration of recombinant human chorionic gonadotropin (rhCG) were comparable between controls and PCOS. Serum E_2_ levels on the day of rhCG administration were high in women with PCOS while P_4_ levels were similar between these groups. Analysis of oocyte parameters revealed that the total numbers of follicles, oocytes (mature + immature), mature MII oocytes, %MII oocytes, and number of fertilized oocytes were unchanged between controls and women with PCOS. However, the rates of fertilization of MII oocytes were low in women with PCOS compared to controls. In serum and follicular fluid (FF) samples collected on the day of oocyte pick up (d-OPU), total testosterone (TT) levels were high and sex hormone binding globulin (SHBG) levels were low in PCOS. Androgen-excess indices such as free testosterone (Free-T), bioavailable testosterone (Bio-T), and free androgen index (FAI) were high in FF, while in serum, only Bio-T and FAI were high in PCOS.

**Table 1:** Clinical characteristics of study participants undergoing controlled ovarian hyperstimulation (COH) assessed before and after oocyte retrieval

Parameters assessed	Control (*n* = 20) median (IQR)	PCOS (*n* = 20) median (IQR)	*P* value
Age in years	30.0 (27.0–31.0)	31.5 (28.0–33.0)	0.124
BMI (kg m^−2^)	23.03 (20.97–25.06)	24.65 (22.24–27.76)	0.173
^#^Basal FSH (μU/mL)	7.64 (5.52–9.39)	5.71 (4.39–6.61)	0.037*
^#^Basal LH (μU/mL)	6.19 (3.0–7.66)	9.13 (5.58–12.24)	0.02*
^#^LH:FSH	0.75 (0.55–1.04)	1.74 (1.15–2.04)	0.0001***
^#^Prolactin (ng/mL)	14.2 (12.0–18.27)	14.48 (11.72–24.3)	0.43
^#^TSH (mIU/mL)	1.8 (1.14–2.75)	2.36 (1.55–4.08)	0.0577
^#^AMH (ng/mL)	3.4 (1.94–7.86)	7.99 (5.21–9.74)	0.0094**
Regular cycle	20 (100%)	3 (15%)	
Oligomenorrhea	0 (0%)	15 (80%)	<0.0001***
Secondary amenorrhea	0 (0%)	2 (5%)	
rFSH (IU)	1600 (1528–2313)	1706 (1350–2025)	0.788
E_2_ (ng/mL) before hCG administration	1.49 (1.247–2.24)	2.03 (1.22–2.47)	0.367
E_2_ (ng/mL) on hCG administration day	1.71 (1.436–2.32)	2.56 (1.82–3.72)	0.038*
P_4_ (ng/mL) before hCG administration	0.3 (0.2–0.55)	0.25 (0.17–0.525)	0.522
P_4_ (ng/mL) on hCG administration day	2.65 (2.07–4.05)	4.6 (1.95–6.32)	0.200
Total follicles (*n*)	15.0 (12.0–20.5)	18.5 (15.0–29.25)	0.099
Total oocytes (*n*)	13.5 (10.0–17.25)	16.5 (14.5–26.75)	0.059
MII oocytes (*n*)	10.5 (8.0–13.5)	15.0 (9.0–19.75)	0.057
MII oocytes (%)	82.84 (70.24–90.71)	86.85 (74.2–95.31)	0.366
Total fertilized oocytes (*n*)	6.0 (4.5–9.25)	6.5 (3.75–14.5)	0.464
Rate of MII oocyte fertilization (ROF)	65.63 (51.92–88.13)	52.27 (34.8–67.)	0.039*
^$^E_2_ (ng/mL) Serum	0.2 (0.15–0.6)	0.43 (0.15–0.76)	0.418
^$^E_2_ (ng/mL) FF	374 (178.6–541.7)	280.3 (125–819)	0.586
^$^P_4_ (ng/mL) Serum	1 (3–9)	3.9 (0.3–7.)	0.903
^$^P_4_ (ng/mL) FF	8170 (4760–15,880)	6000 (3280–10,140)	0.325
^$^TT (ng/dL) Serum	126 (89–178.5)	172.6 (140–311.3)	0.020*
^$^TT (ng/dL) FF	300 (140.2–372.6)	405.8 (222.6–639.3)	0.030*
^$^SHBG (nmol/L) Serum	130 (103.0–150.0)	95 (79–134.5)	0.041*
^$^SHBG (nmol/L) FF	158 (127.8–205.5)	127.7 (103.3–148.8)	0.024*
^$^Free T (pmol/L) Serum	3.53 (2.07–4.85)	6.03 (3.93–9.73)	0.066
^$^Free T (pmol/L) FF	4.24 (1.55–7.87)	7.5 (5.28–11.74)	0.050*
^$^Bio-T (nmol/L) Serum	0.82 (0.48–1.14)	1.09 (0.8–1.9)	0.033*
^$^Bio-T (nmol/L) FF	0.99 (0.58–1.84)	1.75 (1.24–2.74)	0.050*
^$^FAI serum	3.03 (2.38–6.48)	6.03 (3.93–9.73)	0.035*
^$^FAI FF	3.7 (2.32–7.17)	6.93 (5.48–13.42)	0.050*

Data are represented as median (inter-quartile range) for anthropometric and hormonal characteristics compared been controls and women with PCOS using Mann-Whitney *U* tests. Parameters marked with asterisk (#) denote those measured between days 3–5 of the menstrual cycle (early follicular phase) before initiating the controlled ovarian hyperstimulation (COH) procedure. Parameters marked by “$” were measured in sera and follicular fluids obtained on the day of ovum pick up (d-OPU). Menstrual characteristics were assessed using the chi-square analysis. *P* values < 0.05 are considered significant for all statistical tests. *P <  0.05, **P <  0.01, ***P </= 0.0001 have been indicated. *BMI* body-mass index, *E*_*2*_ estradiol, *P*_*4*_ progesterone, *TT* total testosteronem, *SHBG* sex hormone binding globulin, *Bio-T* bioavailable testosterone, *Free-T* free testosterone, *FAI* free androgen index.

### Identification of differentially methylated targets and their gene ontology analysis

**Fig. 1:**
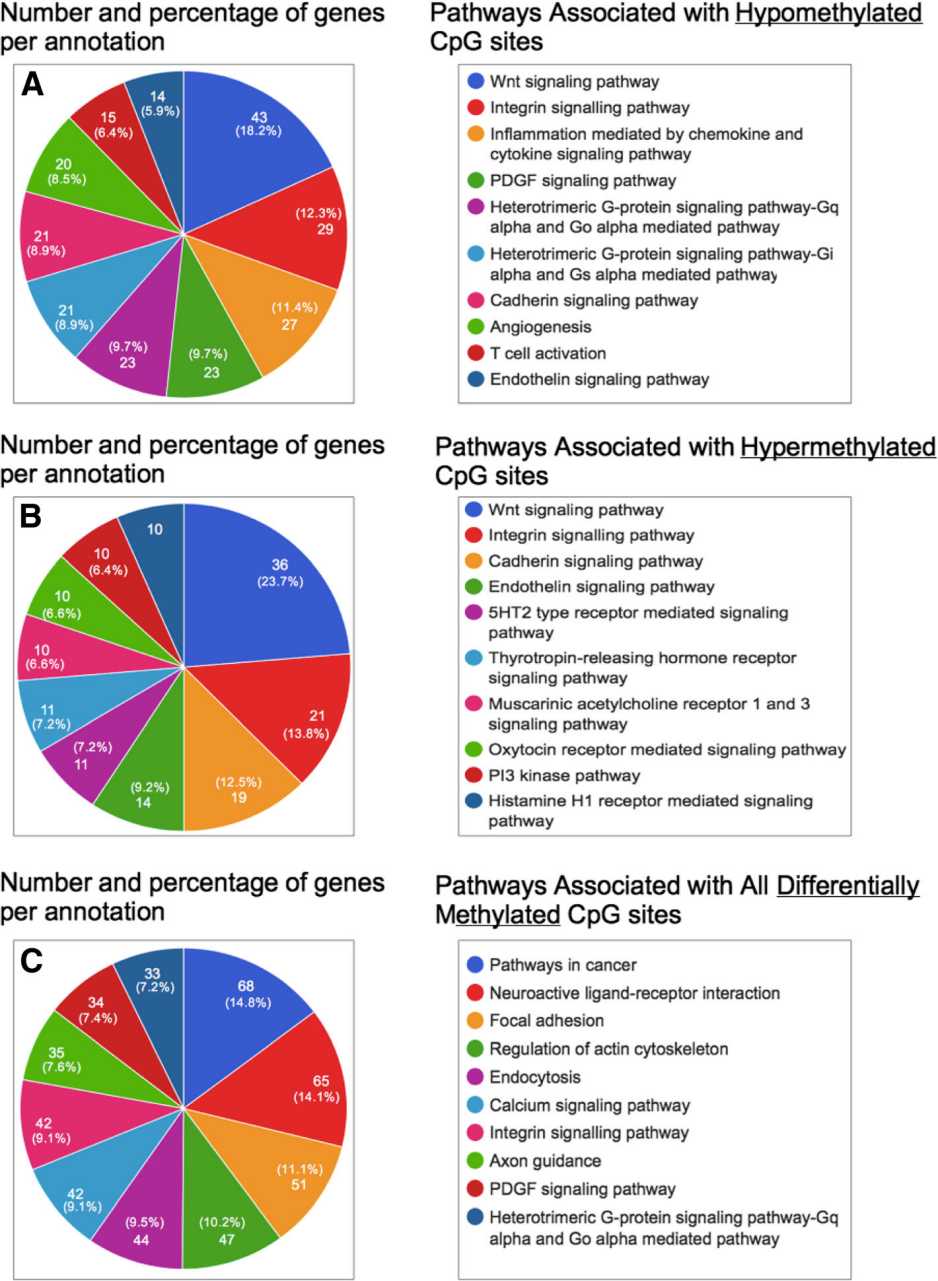
Pathway analysis of differentially methylated genes identified by MC-Seq of cumulus granulosa cells (CGCs). Pie charts displaying gene ontology (GO) and pathway analyses for the lists of hypomethylated genes (**a**), hypermethylated genes (**b**), and all differentially methylated genes (DMGs) (**c**) between CGCs of controls (C-CC, *n* = 3) and women with PCOS (P-CC, *n* = 3) have been shown in the figure. Analysis was carried out using the GeneCodis3 web tool. Each chart represents the top ten pathways enriched in each of the three datasets

MC-Seq of CGCs of women with PCOS and controls identified a total of 6486 differentially methylated CpG sites associated with 3403 unique genes across the genome, of which 2977 CpG sites were hypomethylated and 2509 CpG sites were hypermethylated. Hypomethylated CpG sites were representative of 2063 (Additional file 1) genes in all, while the hypermethylated sites were linked to a total of 1777 genes (Additional file 2). Of the total DMGs, 438 genes harbored both hyper- and hypomethylated CpG sites. Additionally, many noncoding RNAs including 44 microRNAs (miRs) and 121 pseudogenes also showed differential methylation in women with PCOS (Additional files 1 and 2). Of the 44 differentially methylated miRs, several miRs (miR23A, miR127, miR10B, miR193A mir200B, miR182, and miR140) have been reported to be implicated in impaired follicle growth and steroidogenesis, anovulation, obesity, glucose metabolism, and so on [24]. In pathway enrichment and gene ontology (GO) analyses of the hypomethylated, hypermethylated, and combined gene lists, 227 (11%), 210 (11.82%), and 396 (11.64%) genes from the three respective categories could not be annotated. Among the identified pathways, those for Wnt signaling (Panther-GO-ID, P00057), integrin signaling (P00034), endothelin signaling (P00019), and cadherin signaling (P00012) were enriched in both the hypo- and hypermethylated gene sets (Fig. 1). Other prominent pathways included the platelet-derived growth factor (PDGF) signaling (P00047), inflammation mediated by chemokine and cytokine signaling (P00031), angiogenesis (P00005) and vascular endothelial growth factor (VEGF) signaling (P00056), fibroblast growth factor (FGF) signaling (P00021), G protein signaling (P00026, P00027), T cell activation (P00053), and nicotinic acetylcholine receptor signaling pathways (P00044).

### Validation of genes showing differential methylation in regions upstream to transcription start sites

**Fig. 2:**
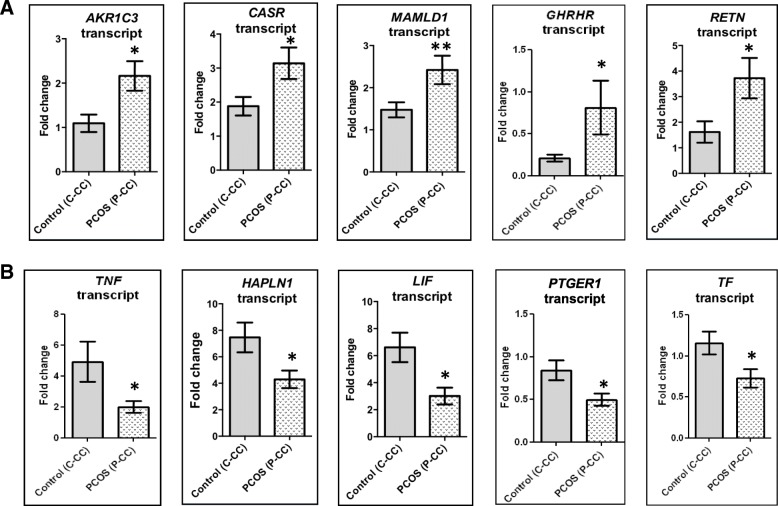
Transcript expression profiling of few differentially methylated genes identified by MC-Seq. Bar graphs representing transcript expression profiles of 10 differentially methylated genes (DMGs) between CGCs (C-CCs) of controls (*n* = 17) and women with PCOS (P-CCs, *n* = 17) are shown in the figure. Panel (**a**) represents transcript expression data of genes identified as hypomethylated in PCOS by NGS analysis while (**b**) shows transcript profiling of hypermethylated genes. Fold change was evaluated using the 2^-ΔΔCt^ method, where the expression was normalized to 18s levels, using a CGC calibrator sample. Data are presented as “mean + SEM.” *P < 0.05, **P < 0.01, 'ns' denotes no significant change. Data are analyzed using the Mann-Whitney *U* test.

**Fig. 3:**
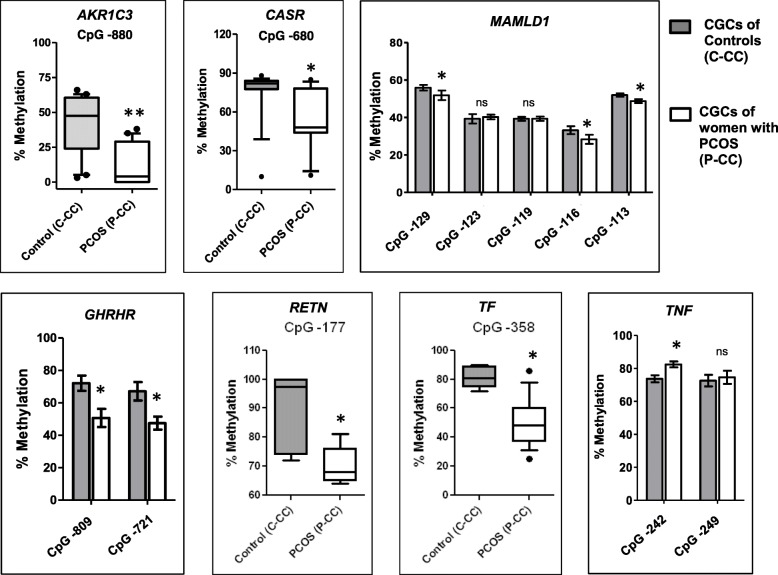
Validation of CpG methylation status of a few differentially methylated genes identified by MC-Seq. The figure depicts “box and whisker” plots for genes whose single CpG site was validated or “bar graphs” for genes in which validation was performed for CpG sites > 1. The plots compare percent (%) methylation for the stated CpG sites between cumulus granulosa cells (CGCs) of controls (C-CC, *n* = 17) and women with PCOS (P-CC, *n* = 17). Data for box plots are presented as whiskers ranging from minimum to maximum values and data for bar graphs are presented as “mean + SEM” using the Mann-Whitney *U* test. *P <  0.05, **P <  0.01, 'ns' denotes no significant change.

Among the DMGs selected for validation, the upstream CpG sites of five genes, namely aldo-keto reductase 1 family C3 (*AKR1C3*), calcium-sensing receptor (*CASR*), growth hormone-releasing hormone receptor (*GHRHR*), resistin (*RETN*), and mastermind-like domain 1 (*MAMLD1*) were hypomethylated while those of transferrin (*TF*) and tumor necrosis factor (*TNF*) were hypermethylated in PCOS in our methylome analysis. We first investigated whether these seven genes are expressed in CGCs, and whether they are differentially expressed in women with PCOS using qPCR (Fig. 2). Apart from *GHRHR*, which was expressed only at baseline levels, the remaining six genes were abundantly expressed in CGCs. Transcripts of *AKR1C3*, *CASR*, *GHRHR*, *RETN*, and *MAMLD1* were upregulated while those of *TF* and *TNF* were downregulated in CGCs of PCOS women (Fig. 2). To verify whether our NGS findings were replicative in a larger study cohort, we performed pyrosequencing to assess the average percent methylation of these genes in 17 controls and 17 women with PCOS. Hypomethylation of *AKR1C3*, *CASR*, *GHRHR*, *RETN*, and *MAMLD1* genes at the indicated CpG sites was confirmed by pyrosequencing (Fig. 3). Decreased methylation in these genes was consistent with the upregulation of their respective transcripts in women with PCOS (Fig. 2), and the two variables showed an inverse correlation with one another (Table 2). Also, hypermethylation of *TNF* was correlated with downregulation of its transcript expression in women with PCOS (Table 2). However, *TF* which showed hypermethylation in NGS data was found to be hypomethylated upon pyrosequencing, though its transcript was downregulated in PCOS (Figs. 2 and 3). Also, there was no correlation between *TF* transcript levels and its methylation status (Table 2). As supporting evidence, we also evaluated the transcript expression of additional genes such as prostaglandin E receptor (*PTGER1*), leukemia inhibitory factor (*LIF*), and hyaluronan and proteoglycan link protein 1 (*HAPLN1*) which showed hypermethylation in NGS analysis. Transcripts of these genes were downregulated in PCOS (Fig. 2).

**Table 2:** Correlation analysis of CpG methylation levels of selected genes with their transcript expression profilesᅟ

Gene name	CpG site/s	*P* value/FDR cutoff	Methylation and transcript status by pyrosequencing and qPCR	Correlation coefficient (*R*^2^); *P* value
AKR1C3	CpG-880	< 0.05	Hypomethylated upregulated	− 0.53; 0.02*
CASR	CpG-680	< 0.05	Hypomethylated upregulated	− 0.74; < 0.0001*
GHRHR	CpG-809	< 0.025	Hypomethylated upregulated	− 0.669; 0.002*
	CpG-721			− 0.501; 0.034
RETN	CpG-177	< 0.05	Hypomethylated upregulated	− 0.603; 0.029*
MAMLD1	CpG-129	< 0.016	Hypomethylated upregulated	− 0.759; < 0.0001*
	CpG-116			− 0.596; 0.007*
	CpG-113			− 0.612; 0.005*
TNF	CpG-242	< 0.025	Hypermethylated downregulated	− 0.582; 0.011*
TF	CpG-358	< 0.05	Hypomethylated downregulated	− 0.162; 0.507

CpG sites that showed significant difference between controls and PCOS in Fig. 3 were analyzed. *P* values < FDR cutoff values are marked as significant (*)

## Discussion

Tissue-specific DNA methylation changes sired by alterations in the environmental or physiological milieu of an individual can bring about significant changes in gene and protein expression, and therefore predispose them to disease development. Alterations in both transcriptome and proteome profiles of ovarian cells/tissues and FF have been previously reported in PCOS [25–28]. With this background information, we had initially conducted a pilot study to screen for the tissue-specific global DNA methylation changes in PBLs and CGCs of controls and women with PCOS. Here, subtle alterations were detected in CpG methylation profiles of long interspersed nucleotide element 1 (LINE1) in PCOS, and these changes were found to be more prominent in CGCs of women with PCOS, compared to PBLs [8]. Since LINE1s are self-replicating transposons and occupy ~ 17% of the human genome, even slight changes in their methylation patterns can be reflective of genomic dysregulation. Therefore, the primary goal of this study was to identify the genome-wide methylation differences in CGCs of women with PCOS, at a single base resolution. GO analysis of the current methylome data revealed that genes regulating cell growth, adhesion, differentiation, proliferation, cell polarity and fate determination, apoptosis, signal transduction, transcription, post-translational modifications, protein binding, metal and nonmetal ion binding, ATP binding, vesicular transport, etc., were differentially methylated in PCOS (Additional file 3). The implications of differential methylation observed in few of these identified genes, which may contribute to hyperandrogenism, defects in COC expansion, oocyte maturation/and ovulation, premature luteolysis, and oxidative stress observed in PCOS, have been discussed here.

### Androgen overproduction

**Fig. 4:**
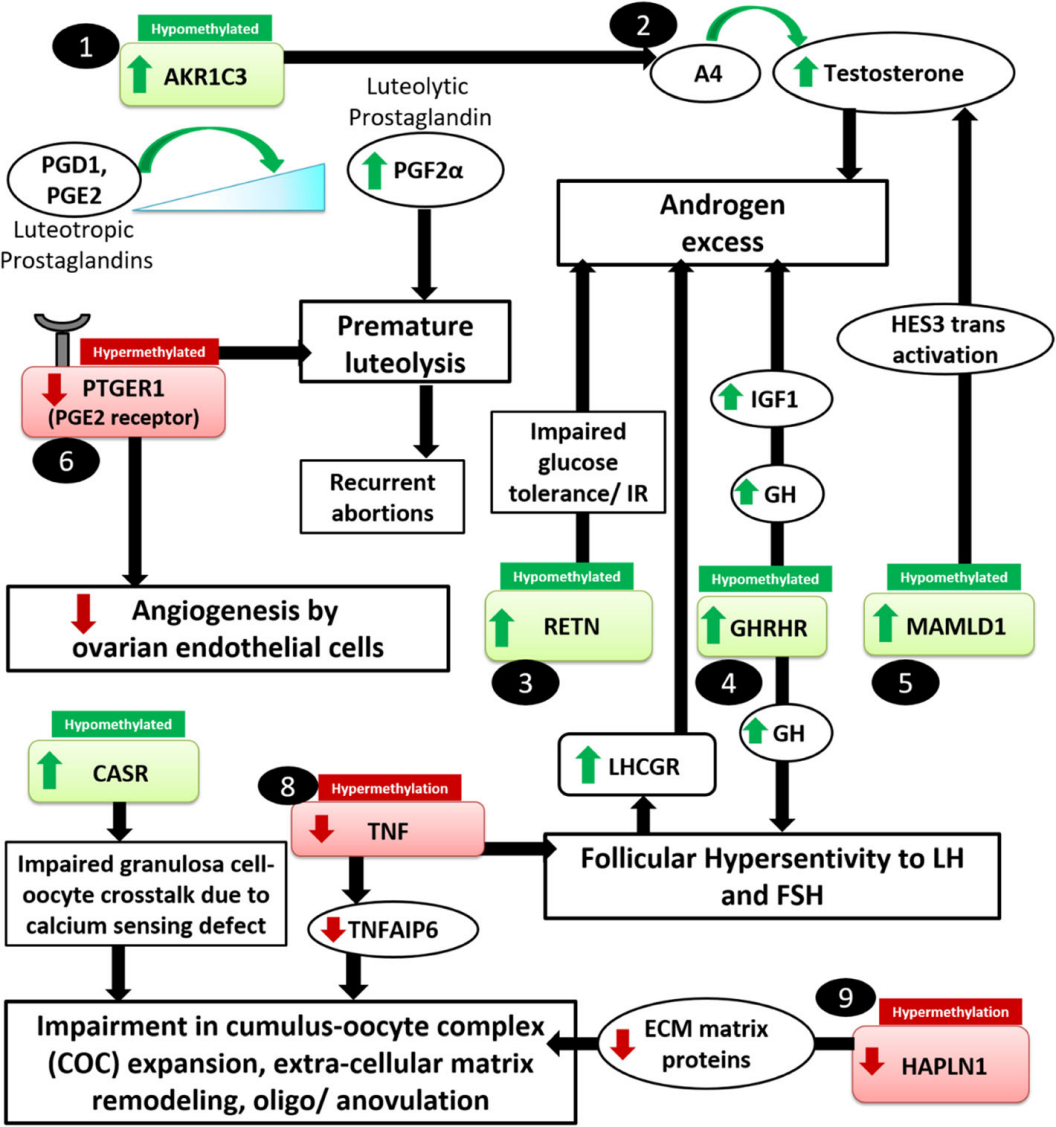
Altered gene methylation can contribute to ovarian dysfuncion. The figure summarizes some of the processes that could be dysregulated in the follicular compartment of women, due to hypomethylation (indicated by green boxes) or hypermethylation (indicated by red boxes) of genes in cumulus granulosa cells (CGCs). AKR1C3, aldo-keto reductase family 1 member C3; AR, androgen receptor; CASR, calcium-sensing receptor; COC, cumulus-oophorus complex; FF, follicular fluid; FSH, follicle-stimulating hormone; FSHR, follicle-stimulating hormone receptor; GC, granulosa cells; IGF1; insulin-like growth factor 1; INS, insulin; MGC, mural granulosa cells; GHRHR, growth hormone-releasing hormone receptor; HAPLN1, hyaluronan and proteoglycan link protein 1; LH, luteinizing hormone; LHCGR, luteinizing hormone chorionic gonadotropin receptor; LIF, leukemia inhibitory factor; MAMLD1, mastermind-like domain containing 1; PTGER1, prostaglandin receptor E1; RETN, resistin; TC, theca cells; TF, transferrin; TNF, tumor necrosis factor alpha

CpG hypomethylation in genes such as *AKR1C3*, *GHRHR*, *MAMLD1* and *RETN,* and hypermethylation in *TNF*, which can indirectly contribute to androgen excess, were consistent with increased and decreased levels of the respective gene transcripts (Figs. 2 and 3). AKR1C3 is a steroidogenic enzyme that converts androstenedione (A4) to biologically active testosterone in non-testicular tissues [29]. In PCOS, the increased expression of AKR1C3 and AKR1C3-mediated androgen production, have been reported in the adrenal cortex and visceral adipose tissues [29, 30]. This enzyme is also expressed by GCs (both mural and cumulus granulosa cells) of periovulatory follicles [31]. Since *AKR1C3* expression was found to be high in CGCs of women with PCOS, it may contribute to the high androgen production in their ovaries. Next, *GHRHR* is a gene encoding the class B GPCR subfamily receptor, which regulates the release of somatotropin (GH) in the brain and other tissues including the ovary, via binding to the hypothalamic neuropeptide GHRH [32, 33]. Although the classical ovarian function of GH is to enhance sexual maturation at puberty via binding to its receptors (GHRs) [34], the GH-GHR interaction also stimulates the release of insulin-like growth factor (IGF1) via transcriptional activation [34]. IGF1, like insulin, can increase LH production from the pituitary and augment ovarian androgen synthesis in PCOS [35, 36]. Additionally, hyperinsulinemia in PCOS is known to increase the bioavailability of IGF1 via the downregulation of its carrier protein, i.e., IGFBP1 [25, 34]. High levels of GH and IGF1 also increase the sensitivity of developing follicles to gonadotropins [37, 38], and PCOS follicles have been reported to exhibit increased sensitivity and responsiveness to FSH [39]. As a result, the expression of LHCGR which is under the direct control of FSH is found to be elevated in the follicles of women with PCOS [13]. This can further augment LH-mediated ovarian androgen production in PCOS. Thus, hypomethylation and overexpression of *GHRHR* observed in CGCs can be an indirect mediator of androgen excess in PCOS (Fig. 4). Further, the pro-inflammatory cytokine TNF, which suppresses FSH-induced *LHCGR* promoter activation via NF-κB p65, was hypermethylated and low in PCOS [40], thus also making it an additional factor contributing to hyperandrogenemia.

Mutations in MAMLD1, a transcriptional coactivator, have been reported to result in compromised androgen synthesis during male fetal sexual development [41, 42]. However, information regarding a definite role of MAMLD1, its regulation and mechanisms of action in the context of other reproductive functions, is limited. In a murine study, *MAMLD1* knockout male mice showed a reduced testosterone production in Leydig tumor cells while the activation of *MAMLD1* promoter by the transcription factor, SF1, augmented testosterone production via transactivation of the hairy/enhancer of split 3 *HES3* promoter [43]. We therefore propose that *MAMLD1* hypomethylation and upregulation of its transcript in CGCs may be important in contributing to androgen excess in PCOS ovaries (Fig. 4). Next, increased circulatory levels of the adipokine, RETN, which modulates glucose tolerance and insulin action, have been linked to a higher incidence of insulin resistance and PCOS [44, 45]. However, there is some ambiguity regarding the role of RETN in PCOS, since its serum and FF levels have been either found to be high or unchanged in PCOS [46–48]. In theca cells, the dose-dependent increase in RETN showed augmented androgen production [49], while a RETN-like molecule β impaired the glucose tolerance and insulin actions in HEK293T cells and adipocytes [50]. Thus, the overexpression of *RETN* due to the hypomethylation of its promoter may be an important factor contributing to the androgen excess in PCOS (Fig. 4).

### Oocyte development, ovulation, and COC matrix expansion defects

Enrichment of calcium (Ca^2+^) signaling pathway and pathways for regulation of cytoskeletal and focal adhesion elements in our NGS analysis indicated impairment of calcium homeostasis and cellular architecture in CGCs of women with PCOS (Fig. 1, Additional file 3). Ca^2+^ signaling pathways are crucial for the development and maturation of healthy oocytes and CASR, which is an important mediator of this pathway, responds to subtle changes in extracellular Ca^2+^ concentrations and activates or ameliorates the mobilization of intracellular Ca^2+^ stored in tissues [51]. Expression and localization of CASR have been reported in human oocytes and CGCs, wherein it supposedly facilitates bidirectional communication between these cells to either keep oocytes arrested in MI phase, or assist in the full resumption of their cytoplasmic and nuclear maturation upon entering the MII phase [52]. Therefore, alterations in CASR expression may affect oocyte maturation and yield poor quality oocytes as seen in PCOS (Fig. 4). In PCOS, so far, a single report exists on the association of a CASR polymorphism (Hin1I) with altered global calcium homeostasis [53]. Altered methylation has been previously reported in human *CASR* promoter in a few cancer conditions [54–56]. Our results indicate that altered *CASR* expression in CGCs of PCOS women can be also influenced by altered methylation.

*TNF* expression in CGCs has been reported to be either unchanged or reduced in CGCs of women with PCOS [57, 58]; however, its circulating levels have been found to be high in their serum and FF [59, 60]. Few studies demonstrated that treatment with high levels of TNF increased GC apoptosis, impaired P_4_ production from GCs, and caused other steroidogenic defects in these cells [61–63]. However, these studies utilized TNF at 10–20-fold higher doses relative to its physiological levels. In alternate studies, optimal TNF levels have been reported to impart a protective function in the maintenance of bovine GCs and oocytes [64], facilitate ovulation [65], and increase the GC proliferation in animal models [66]. Decreased levels of endogenous TNF in GCs has been attributed to diminished oocyte competence due to a reduction in its downstream effector, i.e. tumor necrosis factor-inducible gene 6 (TNFAIP6) [25], as well as compromised ovulation, and GC proliferation in ovarian follicles [67]. Thus, lowered *TNF* in CGCs of women with PCOS owing to hypermethylation, may hamper COC expansion and compromise ovulation. LIF, which was hypermethylated and downregulated in our study, has been reported to be low in FF and serum of women with PCOS [68, 69]. LIF has demonstrated embryotrophic effects in mice and humans [70, 71], and decrease in its levels has shown a positive association with low implantation rates and poor IVF outcome [68, 69]. Since dose-dependent administration of LIF also showed induction of COC expansion and improvement in oocyte competence in humans, it is imperative to investigate the role of epigenetics in the regulation of *LIF* expression in women with PCOS. Our earlier data on proteomics of FF in women with PCOS demonstrated the downregulation of COC matrix proteins including amphiregulin, TNFAIP6, and bikunin, whose diminished expression is implicated in COC matrix expansion defects [25]. Supporting these findings, we also observed hypermethylation and downregulation of *HAPLN1*, which is also a COC matrix-associated protein. HAPLN1 facilitates the expansion of COC matrix and imparts stability to the COC complex by binding to other matrix proteins and proteoglycans like hyaluronic acid, versican, aggrecan, and IαI [72]. Exogenous treatment with HAPLN1 increased the CGC viability in vitro and enabled their transformation into granulosa lutein cells, while knockdown of HAPLN1 decreased the cell viability [72]. Therefore, altered *HAPLN1* methylation may contribute to changes in COC expansion dynamics in PCOS (Fig. 4).

### Luteal insufficiency/premature luteolysis

Apart from being an androgen synthesizing enzyme, AKR1C3 also acts as a prostaglandin F synthase (PGFS), which catalyzes the conversion of the luteotrophic prostaglandins, PGD and PGE_2_, to a luteolytic form, i.e., PGF2α to facilitate luteolysis [29]. Luteal insufficiency and premature luteolysis are frequent occurrences in PCOS [73], and these have been linked to aberrations in angiogenic mechanisms at the ovarian level [74]. Angiogenesis is largely under the control of prostaglandins [75, 76], and AKR1C family of enzymes are some major regulators of prostaglandins [77]. Disparities in levels of pro and anti-angiogenic factors in the ovary are largely responsible for defects in follicle development, premature degeneration of oocytes, and regression of CL due to an inefficient supply of oxygen and nutrients to the growing follicles [74]. Both, our present data and FF proteome study provide compelling evidence supporting angiogenic dysregulation in the ovarian compartment [25]. Since AKR1C3 has been implicated as a trigger for premature luteolysis [78] and was found to be hypomethylated and high in PCOS follicles, it may serve as an important epigenetic target to investigate this phenomenon in PCOS, Till now, no clear evidence exists on whether PGE_2_ or PGF2α levels are altered in PCOS. However, our data shows that the receptor for PGE_2_ gene, i.e., *PTGER1*, was hypermethylated in NGS and its transcript was low in CGCs of women with PCOS. Since activation of PTGER1 by specific agonists has demonstrated increased sprout formation in capillaries, both *in vivo* and *in vitro* [79, 80], it can be an important factor for the restoration of follicular angiogenesis. Also, PTGER1 has been shown to stimulate progesterone biosynthesis in human GCs [81]. Since the follicles of PCOS women lack optimum levels of progesterone required for maintenance of CL, low levels of *PTGER1* in PCOS caused by promoter hypermethylation may explain this shortcoming.

### Oxidative stress

**Fig. 5:**
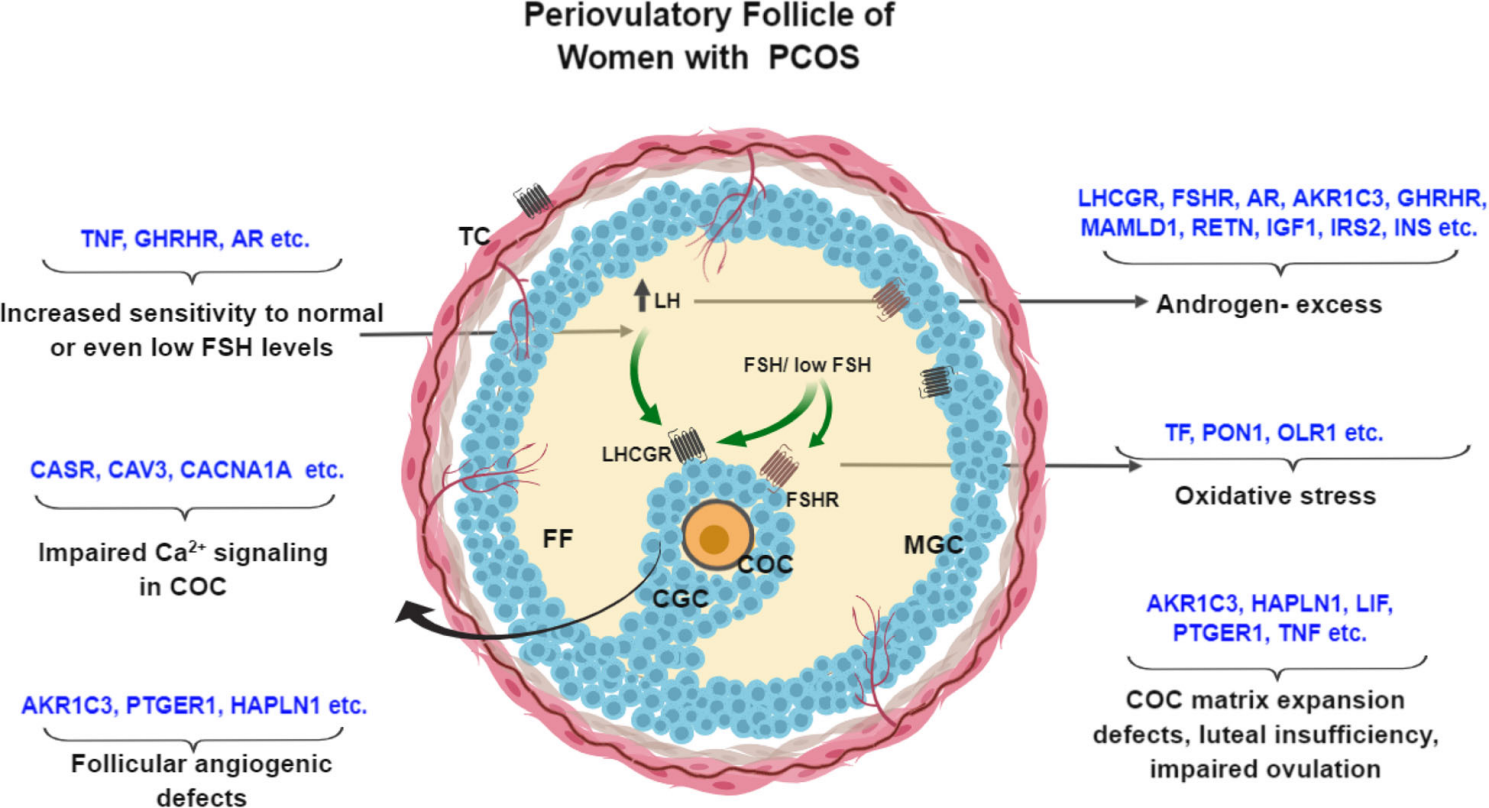
Summary of the genes and their associated processes found to be affected in PCOS due to altered DNA methylation. The figure represents sa preovulatory follicle of women with PCOS, showing a few differentially methylated genes involved in the perpetuation of androgen excess, premature luteolysis, impaired calcium signaling, COC defects, oxidative stress and angiogenic defects in the follicles of women with PCOS as observed in our study. AKR1C3, aldo-keto reductase family 1 member C3; CASR, calcium-sensing receptor; COC, cumulus-oophorus complex; FF, follicular fluid; FSH, follicle-stimulating hormone; FSHR, follicle-stimulating hormone receptor; GC, granulosa cells; IGF1; insulin-like growth factor 1; INS, insulin; MGC, mural granulosa cells; GHRHR, growth hormone-releasing hormone receptor; HAPLN1, hyaluronan and proteoglycan link protein 1; LH, luteinizing hormone; LHCGR, luteinizing hormone chorionic gonadotropin receptor; LIF, leukemia inhibitory factor; MAMLD1, mastermind-like domain containing 1; PTGER1, prostaglandin receptor E1; RETN, resistin; TC, theca cells; TF, transferrin; TNF, tumor necrosis factor alpha

Lastly, *TF*, which maintains the oxido-reductive homeostasis in proliferating cells and showed hypermethylation in our NGS data, was found to be hypomethylated by pyrosequencing, though its mRNA was downregulated in PCOS. Therefore, methylation changes at other CpG sites in *TF* promoter need to be analyzed. TF primarily transports iron released from hepatic, intestinal, and reticuloendothelial stores to the target tissues via its receptor endocytosis while also alleviating local oxidative stress, acting as a growth factor, and promoting follicle and oocyte maturation [82, 83]. Low levels of TF in follicular fluid of PCOS women have been previously reported [84], which may lead to a higher prevalence of unbound Fe^2+^; trigger oxidative damage to DNA, lipids, and proteins; and contribute to oxidative stress, which is reported in PCOS ovaries [85]. Figure 5 depicts a summary of biological processes which may be affected in PCOS due to the altered methylation of genes in follicles of women with PCOS.

### Limitations

The primary limitation of this study was the low sample size. A number of controls and women with PCOS originally recruited for the study had to be excluded due to the presence of confounding factors such as hyperprolactinemia or thyroid dysfunction, and contamination of FF with blood, or due to recent treatment with metformin or thyroid medications. Further, women having low CGC counts were also excluded as the quantities of nucleic acids were insufficient for methylation and expression analyses. Also, since PCOS is a heterogeneous disorder, investigation of epigenetic changes in a large population based on phenotypic subgrouping of women with PCOS (A, B, C, and D phenotypes) as per the Rotterdam consensus may provide more accurate information on the effect of epigenetic components on PCOS development. Due to our limited sample size, it was not possible to carry out such subgrouping and subsequent analyses.

Our findings highlight that MC-Seq could identify several functionally important loci in CGCs, many of which were either known to be functionally dysregulated in PCOS ovary or have a compelling potential to be established as novel candidates influenced by epigenetic changes. The molecular sequelae of these alterations leading to ovarian dysfunction need to be further addressed via robust functional studies in women with PCOS.

## Materials and methods

### Study design, participants, sample collection, and estimated parameters

This study was carried out at the ICMR-National Institute for Research in Reproductive Health (NIRRH) as per ethical norms. All participants were recruited from the “Fertility Clinic and IVF Center” (Mumbai) after obtaining written informed consents and underwent IVF using a long, GnRH agonist protocol as reported earlier [8]. We initially recruited 35 women with PCOS and 38 age-BMI-matched, healthy, and regularly menstruating controls as per the Rotterdam consensus criteria [86]. Women showing normal ovarian morphology on ultrasound with no signs of hyperandrogenism or insulin resistance, and undergoing COH strictly owing to indications of male factor infertility in their spouses were recruited as controls. Women recruited as PCOS had at least 2 of the following 3 features, i.e., polycystic ovaries (PCO) on ultrasound, irregularity/absence of menses, and/or signs of hyperandrogenism during clinical screening, however the presence of PCO morphology was used as a mandate for all women recruited as PCOS. These women were carefully screened for their baseline hormonal estimates and biochemical characteristics using serum and follicular fluid samples (Table 1). Further, we had to exclude samples of women who were on metformin or thyroid medications, whose follicular fluid had blood contamination and whose CGC counts were low, since substantial amounts of DNA were required for high-throughput methylome sequencing and bisulfite PCRs. After this, 20 controls and 20 women with PCOS were selected for the study (Table 1). Baseline hormonal estimates (between days 3–7 of menstrual cycle) for LH, FSH, prolactin, TSH, and AMH could be obtained from IVF clinical records, while fasting serum and macroscopically clear FF collected on d-OPU were assayed for E_2_, P_4_, TT, and SHBG using commercial ELISA kits (Diagnostics Biochem Canada Inc., Dorchester, Ontario, Canada). Androgen excess indices were calculated using TT and SHBG values [8]. Upon follicle maturation, the levels of E_2_ and P_4_, which are routinely measured 1 day prior to and 1 day after rhCG administration (10,000 IU) to monitor ovarian response, were also recorded. COCs suspended in FF aspirates were separated from FF and manually stripped off to dissociate the cumulus granulosa cells (CGCs) from their oocytes. CGCs were washed, resuspended in ovum buffer, and transported from IVF center to the lab at 37 °C for further processing. The numbers of total retrieved oocytes and mature oocytes (in the MII phase of meiosis) were obtained from clinical records, and rates of fertilization of MII oocytes were calculated.

### Methyl-capture sequencing (MC-Seq) by NGS approach and analysis of DMRs

From among the 20 women with PCOS and 20 controls selected for the study, individual DNA samples of CGCs from 3 PCOS women and 3 age-BMI-matched controls having a total yield of > 2 μg were subjected to MC-Seq using the NGS approach. Whole genomic DNA was extracted from CGCs of these women using QIAamp DNA mini kit (Qiagen, Hilden, Germany) and processed for library preparation. 1 μg of genomic DNA per sample was sheared in fragments of approximately 150 bp using the Covaris S220 ultrasonicator and further processed for DNA end repair, 3′-adenylation, methylated adapter ligation, and hybridization to the Methyl-Seq library probes using the SureSelect Methyl-Seq Library Prep Kit (Agilent Technologies, CA, USA) as per the manufacturer’s instructions. The hybrids were captured using streptavidin beads (Dynabeads) and subjected to bisulfite conversion using the EZ-DNA Methylation Gold Kit (Zymo Research, CA, USA). The library was PCR amplified, indexed using SureSelectXT Methyl-Seq indexing primers (Agilent Technologies, CA, USA), and pooled for 100 bp paired-end multiplexed sequencing on Illumina HiSeq 2500 platform.

### Processing and alignment of reads

Whole-genome targeted methylation sequencing reads of the above six samples were aligned to human reference genome hg19 assembly. The hg19 reference genome was converted to a DNA methylation reference genome, and genome indexing was performed using Bismark genome preparation utility (v.14.3). The adapter trimmed sequencing reads were aligned to the converted methylation reference genome using Bismark tool with two allowed mismatches. The remaining parameters from Bismark were used as default. Further, deduplication of aligned reads was performed using “deduplicate_bismark” utility of Bismark tool.

### Methylation analysis and functional annotation

Methylation extractor utility of Bismark tool was used to extract the methylation call for every methylated cytosine (C) in all three contexts: CpG, CHG, and CHH (where H is A or C or T). Differential methylation analysis was performed using “calculateDiffMeth” function of R-based methylKit package at *q* value cutoff threshold of ≤ 0.01 and methylation difference ≥ 25%. Hierarchical clustering of samples based on the similarity of their methylation profile was performed using the euclidean distance metric, and ward method clustering approaches of methylKit package. Further, enrichment and annotation of both hypo- and hypermethylated sites within up 10 kb of annotated transcription start site (TSS) to transcription end site (UCSC hg19) were performed using in-house perl scripts. GeneCodis3 web-based tool [87] was used for gene ontology (GO) and pathway enrichment analysis of the DMGs.

### Selection of genes for validation studies

Genes showing differential methylation at CpG sites upstream up to 1000 bases relative to their TSS in our NGS data were selected for validation. These included a total of 354 hypermethylated and 397 hypomethylated genes (total *n* = 735 unique genes). The lists of these 735 genes, and the 4354 genes enlisted in Ovarian Kaleidoscope (OKdb), were compared using a Venn analysis, and a total of 132 genes that were common to both datasets could be identified. OKdb is an online search tool based on microarray-based transcriptome profiling and independent study reports on genes and proteins identified in ovarian tissues and cells [88]. Upon identification of genes common to our NGS dataset and OKdb, and a careful review of literature, 7 genes participating in the perpetration of androgen excess, impaired angiogenesis, luteal insufficiency, COC matrix defects, and oocyte defects, namely *AKR1C3, CASR, GHRHR, MAMLD1, RETN, TF,* and *TNF*, were selected for validation by both pyrosequencing and qPCR. Additionally, the transcript expression profiles of prostaglandin E receptor (*PTGER1)*, leukemia inhibitory factor *(LIF)*, and hyaluronan and proteoglycan link protein 1 (*HAPLN1*), were evaluated in CGCs of controls and PCOS women as supporting evidence for pathways associated with the above genes.

### Extraction of nucleic acids from CGCs for qPCR and pyrosequencing

Total DNA and RNA were extracted from CGCs of 17 controls and 17 women with PCOS using the NucleoSpin TriPrep kit (Macherey-Nagel, Düren, Germany) for validation of the NGS data. The quality and yield of nucleic acids were assessed by agarose gel electrophoresis and by evaluating their spectrophotometric ratios at 260 and 280 nm. DNA samples were stored at − 20 °C while RNA was stored at − 80 °C until further use.

### cDNA synthesis and quantitative real-time PCR

cDNA was synthesized from 500 ng of RNA (*n* = 34) using a first strand cDNA synthesis kit (Takara Bio USA Inc.). Transcript levels of DMGs selected for validation (Additional file 4) were assayed by TaqMan chemistry using the TaqMan™ Universal Master Mix II with UNG, and FAM-labeled probes (ThermoFisher Scientific, MA, USA). Assay containing VIC-labeled 18s rRNA probe was used as the housekeeping control. qPCR was carried out using cDNA dilutions ranging between neat to 1:100. Fold change in gene expression between controls and PCOS was evaluated using the 2^-ΔΔCt^ method, where the expression was normalized to 18s levels, using a CGC calibrator sample.

### Bisulfite primer design and pyrosequencing

Primers for the validation of selected genes were designed on the Pyromark Q96 ID machine using the PyroMark® Assay Design SW 2.0 software (Qiagen), the list of which has been provided in Additional file 4. Primers were procured from Sigma-Aldrich. The reverse pyrosequencing primers were tagged with biotin at the 5′-end and HPLC purified. Approximately 300–500 ng DNA from CGCs of 17 controls and 17 women with PCOS was bisulfite converted using the MethylCode bisulfite conversion kit (Invitrogen-ThermoFisher Scientific, MA, USA). The region of interest was amplified by two rounds of PCR. PCR product (1–3 μL from total volume of 20 μL) from the first round was used as template for the 2^nd^ PCR round, which was scaled up to 45 μL. For both PCRs, initial denaturation was performed at 95 °C for 15 min followed by 40 rounds of amplification at 94 °C for 30s, annealing at the respective optimized temperatures for 10s, and 72 °C for 60s with a final extension at 72 °C for 10 min. Product amplification after both rounds was confirmed by agarose gel electrophoresis. For pyrosequencing, 40 μL of the PCR product was subjected to clean up on the Pyromark Q96ID sequencing workstation as per the manufacturer’s instructions and sequenced using 1.6 μL (16 picomoles) of each of the gene-specific sequencing primers. 

### Statistical analysis

Mann-Whitney *U* tests were employed for all univariate assessments of continuous variables including hormonal and biochemical parameters, DNA methylation, and transcript expression levels assessed between CGCs of controls and women with PCOS. Correlation between the CpG methylation status of genes and their respective transcripts was determined using a two-tailed Spearman’s correlation coefficient. FDR cutoffs were applied for genes showing multiple CpG sites as significant after Mann-Whitney testing.

## Additional files

Additional file 1: Hypomethylated CpG sites were representative of 2063 genes. (XLSX 408 kb)

Additional file 2: Hypermethylated sites were linked to a total of 1777 genes (XLSX 354 kb)

Additional File 3: Figure includes horizontal bar charts showing components such as A) molecular functions, B) biological processes, and C) cellular components that were most highly enriched in datasets obtained for all differentially methylated, hypomethylated, and hypermethylated genes identified in the NGS analysis. *X*-axis represents the number of genes present within each annotated category. (TIF 901 kb)

Additional File 4: The table enlists pyrosequencing primer sets designed to evaluate upstream/promoter CpG methylation in differentially methylated genes (DMGs) selected for validation in controls (C-CC, *n* = 17) and women with PCOS (P-CC, *n* = 17) and commercial TaqMan Assay IDs used for validating the transcript expression levels of selected DMGs in controls and women with PCOS. (DOCX 13 kb)

## Abbreviations

A4: Androstenedione
AKR1C3: Aldo-keto reductase family 1 member C3
AMH: Anti-Mullerian hormone
AMHR2: Anti-Mullerian hormone receptor 2
AR: Androgen receptor
CASR: Calcium-sensing receptor
C-CC: Cumulus granulosa cells of controls
CGC: Cumulus granulosa cell
CL: Corpus luteum
COC: Cumulus-oophorus complex
COH: Controlled ovarian hyperstimulation
DMG: Differentially methylated gene
E_2_: Estradiol
EDC: Endocrine disruptor
FAI: Free androgen index
FF: Follicular fluid
FGF: Fibroblast growth factor
FSH: Follicle-stimulating hormone
FSHR: Follicle-stimulating hormone receptor
FST: Follistatin
GC: Mural and cumulus granulosa cells
GH: Growth hormone
GHR: Growth hormone receptor
GHRHR: Growth hormone-releasing hormone receptor
GnRH: Gonadotropin-releasing hormone
GO: Gene ontology
H6PD: Hexose-6-phosphate dehydrogenase/glucose 1-dehydrogenase
HAPLN1: Hyaluronan and proteoglycan link protein 1
hCG: Human chorionic gonadotropin
IGF1R: Insulin-like growth factor receptor
IGFBP: Insulin-like growth factor binding proteins
INS: Insulin
IRS-2: Insulin receptor substrate 2
IVF: In vitro fertilization
LH: Luteinizing hormone
LHCGR: Luteinizing hormone chorionic gonadotropin receptor
LIF: Leukemia inhibitory factor
LINE1: Long interspersed nucleotide element 1
MAMLD1: Mastermind-like domain containing 1
MC-Seq: Methyl-capture sequencing
MeDIP: Methylated DNA immunoprecipitation
MGC: Mural granulosa cells
NGS: Next generation sequencing
OKdb: Ovarian Kaleidoscope
P_4_: Progesterone
PBL: Peripheral blood leukocytes
P-CC: Cumulus granulosa cells of PCOS
PCOS: Polycystic ovary syndrome
PDGF: Platelet-derived growth factor
PGD: Prostaglandin D
PGE2: Prostaglandin E2
PGF: Prostaglandin F
PGFS: Prostaglandin F synthase
PRKCB: Protein kinase C Beta
PTGER1: Prostaglandin receptor E1
RETN: Resistin
SHBG: Sex hormone binding globulin
T2DM: Type 2 diabetes mellitus
TES: Transcription end site
TF: Transferrin
TNF: Tumor necrosis factor alpha
TNFAIP6: Tumor necrosis factor-inducible gene 6
TSH: Thyroid stimulating hormone (TSH)
TSS: Transcription start site
TT: Total testosterone
VEGF: Vascular endothelial growth factor
WGBS: Whole genome bisulfite sequencing
YAP1: Yes-associated protein 1
