# Supplementary figures and images for: DNA methylome profiling of granulosa cells reveals altered methylation in genes regulating vital ovarian functions in polycystic ovary syndrome
Source: Clin Epigenetics. 2019 Apr 11;11:61. doi: 10.1186/s13148-019-0657-6 (PMC6458760; doi:10.1186/s13148-019-0657-6)

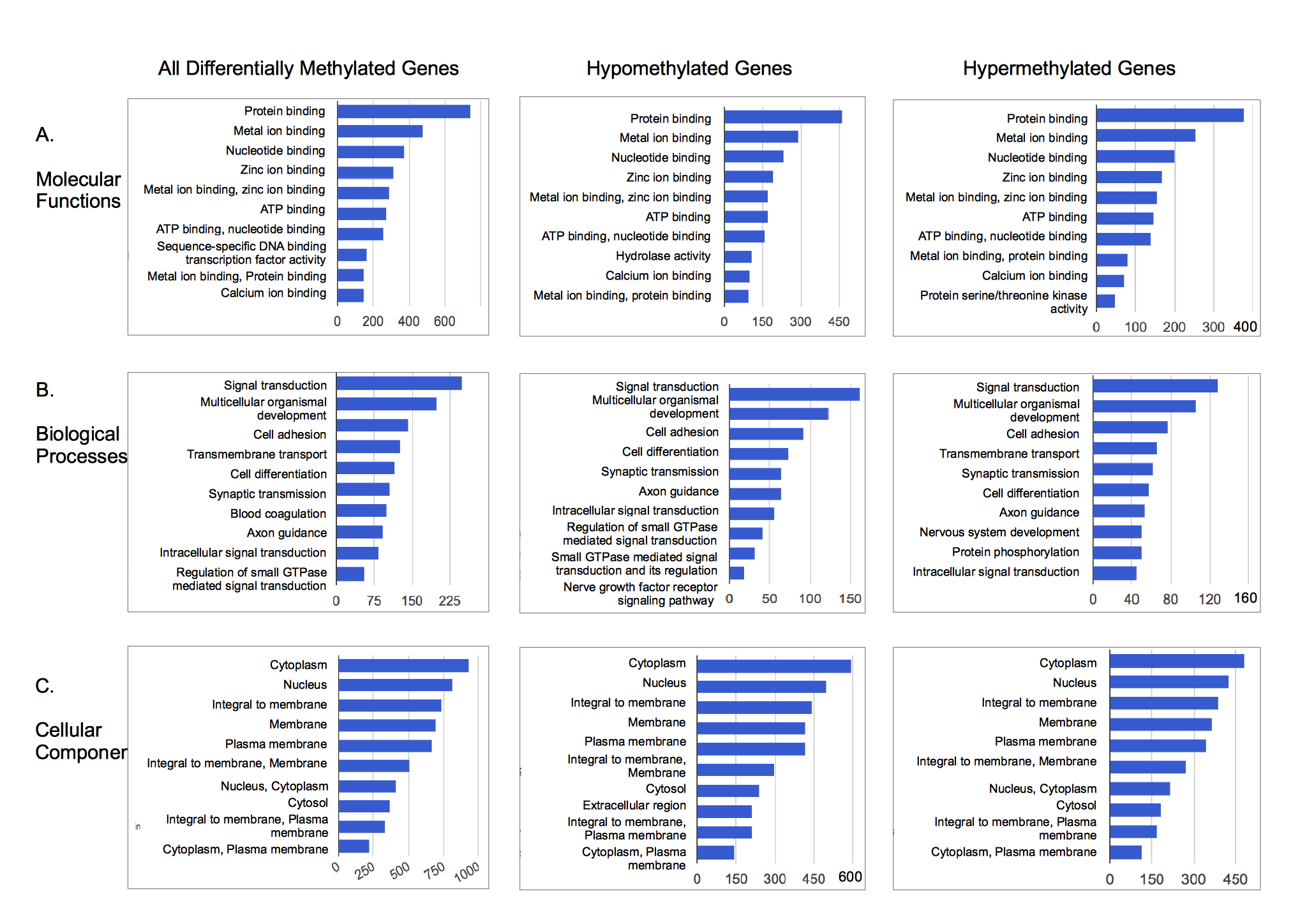

Supplement: Supplementary file 3 — Figure includes horizontal bar charts showing components such as A) molecular functions, B) biological processes, and C) cellular components that were most highly enriched in datasets obtained for all differentially methylated, hypomethylated, and hypermethylated genes identified in the NGS analysis. X-axis represents the number of genes present within each annotated category. (TIF 901 kb) [file 13148_2019_657_MOESM3_ESM.tif]
