# Supplementary material for: DNA methylome profiling of granulosa cells reveals altered methylation in genes regulating vital ovarian functions in polycystic ovary syndrome
Source: Clin Epigenetics. 2019 Apr 11;11:61. doi: 10.1186/s13148-019-0657-6 (PMC6458760; doi:10.1186/s13148-019-0657-6)
Supplement: Supplementary file 4 — The table enlists pyrosequencing primer sets designed to evaluate upstream/promoter CpG methylation in differentially methylated genes (DMGs) selected for validation in controls (C-CC, n = 17) and women with PCOS (P-CC, n = 17) and commercial TaqMan Assay IDs used for validating the transcript expression levels of selected DMGs in controls and women with PCOS. (DOCX 13 kb) [file 13148_2019_657_MOESM4_ESM.docx]

**Supplementary Table**

| **Gene Symbol** | **Primers for Pyrosequencing** | | | **TaqMan assay probes for transcript expression** |
| --- | --- | --- | --- | --- |
|  | **Forward Primer**  **(5’ – 3’)** | **Reverse Primer**  **(Biotin-5’ – 3’)** | **Sequencing Primer (5’ – 3’)** |  |
| AKR1C3 | TGAGGATTTTATGTGTTTATTGTTAGT | ACTTATTTCTACCTTTACAAAACAATTAA | TGTTGTTTTATTAAAAAGTAAATGG | Hs00366267_m1 |
| CASR | CACAAAAAACAAACAAAACCAAACTATC | TTTAAGGATTTTTTGAGGTAGATGT | CAAATAAAAATACAAATTTCTTCTC | Hs01047795_m1 |
| GHRHR Set1 | AACCATCTTAAATAAAAAAAAAACAACTCT | AGTTTGGTTAATGGAGATTTTGTAT | CTTAAATAAAAAAAAAACAACTCTC | Hs01081591_m1 |
| GHRHR Set2 | CATTTCAACAAAACCTAAATCCTTTACT | TGTTTTTAGTTTGGTTAATGGAGATT | ACTTATCAAAATCTCTCCAA |  |
| MAMLD1 | GGAGTGGAAATTTTGAGTTTTTAGG | CACACTCACACACCCTCA | AGGGTAGAGAGGTTTTTTTTTTAAG | Hs00193976_m1 |
| RETN | AAACCTCTTAAAATAAAACTTCCTAACTT | GGTATTATTTTTGGGGTATTATTTTTTGAT | TCCCACTTCCAACAA | Hs00220767_m1 |
| TF | GTTGGAGGGAGTTTATTTTTTTTTATGATT | ACCTTTTACTCCCCACCAAATA | AGTTTAAGGTGTTTTATAGG | Hs00169070_m1 |
| TNF-α | TGTTTGGAAGTTAGAAGGAAATAGA | AAAATACCCCTCACACTCC | AGTTAGTGGTTTAGAAGAT | Hs00174128_m1 |
| LIF | - | - | - | Hs01055668_m1 |
| HAPLN1 | - | - | - | Hs00157103_m1 |
| PTGER1 | - | - | - | Hs00909194_g1 |
